# Migratory Connectivity of the Monarch Butterfly (*Danaus plexippus*): Patterns of Spring Re-Colonization in Eastern North America

**DOI:** 10.1371/journal.pone.0031891

**Published:** 2012-03-13

**Authors:** Nathan G. Miller, Leonard I. Wassenaar, Keith A. Hobson, D. Ryan Norris

**Affiliations:** 1 Department of Integrative Biology, University of Guelph, Guelph, Ontario, Canada; 2 Environment Canada, Saskatoon, Saskatchewan, Canada; The Australian National University, Australia

## Abstract

Each year, millions of monarch butterflies (*Danaus plexippus*) migrate up to 3000 km from their overwintering grounds in central Mexico to breed in eastern North America. Malcolm et al. (1993) articulated two non-mutually exclusive hypotheses to explain how Monarchs re-colonize North America each spring. The ‘successive brood’ hypothesis proposes that monarchs migrate from Mexico to the Gulf Coast, lay eggs and die, leaving northern re-colonization of the breeding range to subsequent generations. The ‘single sweep’ hypothesis proposes that overwintering monarchs continue to migrate northward after arriving on the Gulf coast and may reach the northern portion of the breeding range, laying eggs along the way. To examine these hypotheses, we sampled monarchs throughout the northern breeding range and combined stable-hydrogen isotopes (δD) to estimate natal origin with wing wear scores to differentiate between individuals born in the current vs. previous year. Similar to Malcolm et al. (1993), we found that the majority of the northern breeding range was re-colonized by the first generation of monarchs (90%). We also estimated that a small number of individuals (10%) originated directly from Mexico and, therefore adopted a sweep strategy. Contrary to Malcolm et al. (1993), we found that 62% of monarchs sampled in the Great Lakes originated from the Central U.S., suggesting that this region is important for sustaining production in the northern breeding areas. Our results provide new evidence of re-colonization patterns in monarchs and contribute important information towards identifying productive breeding regions of this unique migratory insect.

## Introduction

Despite the massive scale of some insect migrations, remarkably little is known about how populations are spatially connected between different periods of the annual cycle (i.e. migratory connectivity) [1], [2]. Most migratory insects (e.g. dragonflies, butterflies, milkweed bugs) require multiple generations to complete a single annual migratory cycle [3]. This, along with the fact that small, short-lived organisms are almost impossible to track using conventional external markers, has greatly hindered progress in understanding migratory patterns [2]. Nevertheless, knowledge of the degree of connectivity between stages of the migratory cycle is critical for predicting changes in population size [4] and for developing effective conservation strategies [5].

Nearly the entire eastern North American population of monarch butterflies (*Danaus plexippus*) migrate thousands of kilometres southward to discrete overwintering sites located between 2400–3600 m.a.s.l. in the Oyamel forests of central Mexico [6], [7]. However, the strategy that monarchs use to recolonize eastern North America each spring remains largely a mystery. In 1878, Edwards proposed that re-colonization of the eastern breeding region was accomplished by the first spring generation of monarchs [8], whereas in 1881, Scudder suggested that monarchs leaving the eastern breeding region in the fall return during the spring to produce offspring [9]. With the discovery of the overwintering sites, Malcolm et al. [10] modified these hypotheses. The ‘successive brood’ hypothesis (hereafter termed “SB strategy”) proposes that overwintering monarchs migrate north to the U.S. Gulf Coast, lay eggs, and die, leaving the re-colonization of the Great Lakes region to the 1^st^ spring generation. The ‘single sweep’ hypothesis (hereafter termed “SS strategy”) proposes that re-colonization is accomplished by the overwintering generation arriving at the Gulf Coast in early spring to lay eggs, but then continuing northward towards the Great Lakes, effectively recolonizing the entire eastern breeding range in a ‘single sweep’.

Using cardenolide signatures of a milkweed species (*Asclepias viridis*) associated with the southern U.S.A., Malcolm et al. [10] estimated that 90% of monarchs caught in the spring in the Great Lakes region followed a SB strategy. Although, the geographic range of *A. viridis* is extensive and reaches as far north as Ohio [11], some individuals that were assigned to have followed a SB strategy had very worn wings. Thus, these results don't preclude the possibility that some of these individuals could have been born north of the Gulf Coast the previous year and completed a return migration from Mexico (i.e. followed a SS strategy). Importantly, although Malcolm et al. [10] speculated that the remaining 10% of monarchs they sampled followed a SS strategy, they did not have sufficient concentrations of cardenolides in monarch tissues to determine the larval foodplant of these individuals [10].

**Figure 1:**
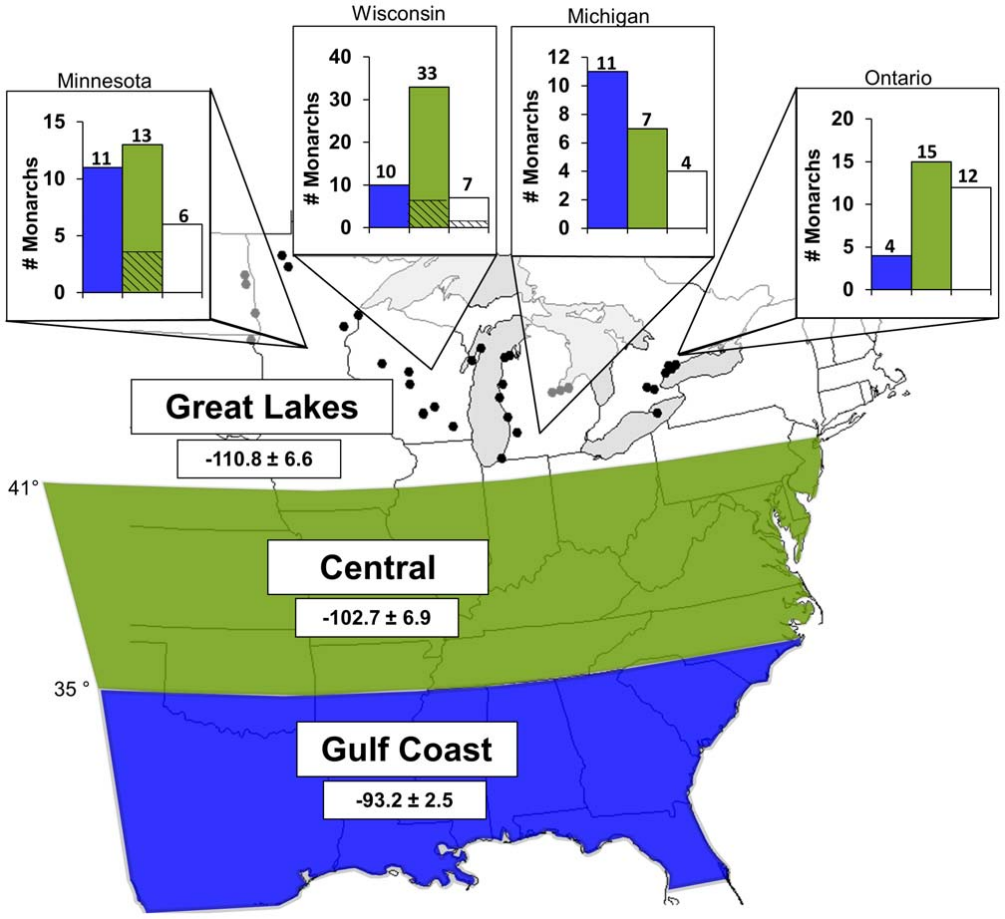
The estimated natal origin of monarchs captured in the Great Lakes region. Bar graphs (number above each bar shows sample size) indicating the number of monarchs originating from one of three breeding regions: Gulf Coast (blue), Central (green), and the Great Lakes (white). Bar graphs are arranged according to the state/province where monarchs were sampled (black dots indicate sampling locations). Bars with cross-hatching represent the proportion of monarchs estimated to have followed a SS strategy, all others are SB (blue and green non-hatched) or the offspring of SS/SB monarchs (white bars). Numbers within each breeding region are the mean ± SD stable-hydrogen isotope (δD) values calculated from precipitation and adjusted according to a fixed discrimination factor (see methods for details on assignment tests).

Here, we examined these two re-colonization hypotheses by sampling monarchs from 44 sites across the Great Lakes region (fig. 1). To distinguish whether adults arriving at the northern part of their breeding range employed a SS (over wintering generation that are born the previous year) or SB (1^st^ generation born in current year) strategy, we used stable-hydrogen isotopes (δD) to estimate natal origin [12] and wing wear to estimate age (see table 1 for predictions).

**Table 1:** Summary of predictions (first two rows) to distinguish whether monarchs arriving in northern portion of the breeding range adopt a single sweep (SS) or successive brood (SB) strategy.

Natal Origin based on Stable Isotopes^1^	Great Lakes (GL)		Central (CE)		Gulf Coast (GC)	
Year Born based on Wing Wear^2^	Previous	Current	Previous	Current	N/A^2^	N/A^2^
Inferred Strategy	SS	SS/SB^3^	SS	SB	SB	

Notes:

1 See Figure 1 for information on stable-hydrogen isotope values between regions and Methods for information on assignment tests.

2 Individuals with wing wear scores between 1–3 were considered to have been born in the sampling year while individuals with wing wear scores of 4 or 5 were considered to have been born the previous year (see Methods for details). The only exception to this is for individuals that were assigned to the GC region. In this case, all individuals must have been born the sampling year regardless of wing wear because it is unlikely that GC monarchs overwinter in Mexico because milkweed does not grow in the Gulf Coast during late summer when the migratory generation is produced (see Discussion for details).

3 Monarchs born in the GL region in the year they were sampled could be either the offspring from monarchs migrating north from over wintering sites in Mexico (SS) or from monarchs that were born the GC or CE regions the same year (SB).

## Materials and Methods

### Monarch sampling and wing wear

Monarch adults were collected from May through June 2009 throughout the Great Lakes region: Ontario (*n* = 31; 14 sites), Michigan (*n* = 22; 12 sites), Wisconsin (*n* = 50; 11 sites), and Minnesota (*n* = 30; 8 sites; fig. 1, see table 2 in supporting information for full list of sampling sites). Collection of monarchs within the U.S did not occur on protected lands and did not require any specific permits (monarchs are not a protected species within the U.S). Within Ontario, monarch collection was conducted within conservation areas with a Wildlife Scientific Collector's Authorization issued by the Ontario Ministry of Natural Resources (2009). Individuals were captured using standard sweep insect nets and each individual was given a wing wear score based on the classification developed by K. Oberhauser, University of Minnesota (http://monarchlab.umn.edu/lab/research/topics/vitalstats/howtomeasure.aspx) and used by Malcolm et al [10] to provide an approximate estimate of monarch age (born during the current year: SB or the previous year: SS). Scores ranged from 1–5, where 1 = newly emerged individual, 2 = few scales lost, very little fraying on wing edges, 3 = some scales missing, wings becoming dull, some fraying on wing edges, 4 = large numbers of scales missing, substantial tearing around wing margins, 5 = more than a third of scales missing, wings tattered and pieces of wing missing from orange wing cells. We used this score to distinguish between SS individuals (as much as 9 months old) and SB individuals (born during the current sampling year). We classified SS adults as having a score of ≥4 and SB individuals <4 based on evidence from Malcolm et al. [10], who found that monarchs arriving on the Gulf Coast (GC) in early spring from Mexico already had wing wear scores of 3.28 (±0.83). Thus, our wing wear classification was conservative because individuals originating in Mexico must travel an additional ∼1000 km to reach the Great Lakes (GL) region. Furthermore, our results indicated that individuals born in the GC region (estimated from stable-hydrogen isotope values) and captured in the GL region had wing wear scores of 2.94 (±1.16), suggesting that there was a significant increase in wing wear as a result of northward migration between these two regions. Monarchs born in the central (CE) region during the sampling year (i.e. SB strategy) are likely to have even lower wing scores than individuals born in the GC region due to a considerably shorter migration distance (as much as 650 km shorter) and younger age (eggs from the GC region would be laid first), further minimizing the likelihood of confusing SB with SS individuals.

**Table 2:** Monarch sampling sites throughout the Great Lakes region from June 2 to June 30, with information on sampling location (state/province, nearest city, and GPS coordinates), dates, and sample size.

state/province	nearest town	Lat (N)/Long (W)	date sampled	sample size (*n*)
MI	Bay City	43° 40.2/83° 54.7	June 9	1
MI	Unionville	43° 42.3/83° 31.6	June 9	1
MI	Cedar	44° 55.0/85° 49.7	June 10	1
MI	Empire	44° 51.3/86° 02.1	June 10	8
MI	Traverse City	44° 57.6/85° 30.9	June 10	1
MI	Free Soil	44° 4.7/86° 15.6	June 11	2
MI	Mears	43° 40.8/86° 28.4	June 11	1
MI	Grand Haven	43° 02.3/86° 12.4	June 12	3
MI	St. Joseph	41° 49.6/86° 38.8	June 12	1
MI	Allegan	42° 32.8/85° 54.5	June 12	1
MI	Midland	43° 37.0/84° 15.1	June 25	1
MI	Unionville	43° 40.9/83° 31.6	June 25	1
WI	Dousmann	42° 55.8/88° 29.5	June 13	23
WI	Devil	43° 33.8/89° 11.3	June 14	2
WI	Merimac	43° 23.7/89° 40.5	June 14	2
WI	Ellison Bay	45° 14.3/86° 60.0	June 15	5
WI	Marshfield	44° 42.2/90° 11.1	June 16	2
WI	Babcock	44° 19.2/90° 10.7	June 16	4
WI	Chippewa Falls	44° 58.9/91° 18.9	June 17	12
MN	Finlayson	46° 10.2/92° 51.9	June 18	8
MN	Moorhead	46° 36.9/96° 45.2	June 20	5
MN	Goodridge	48° 20.4/95° 31.8	June 19	14
ND	Grand Forks	47° 29.0/97° 9.8	June 20	3
ON	Whitby	43° 50.4/78° 58.1	June 13	3
ON	Windsor	42° 16.5/83° 1.4	June 22	2
ON	Port Rowan	42° 31.1/80° 04.0	June 9–June 22	7
ON	Toronto	43° 39.6/79° 27.4	June 2–June 30	15
			total	133

### Stable-hydrogen isotope analysis

Stable-hydrogen isotope (δD) analysis of wing chitin was conducted at the stable isotope laboratory at Environment Canada, Saskatoon, SK. Captured monarchs were euthanized and stored in paper envelopes. A small section of wing tissue was clipped from the hind wing of each monarch (350±10 µg) and encapsulated in 4.0×3.2 mm silver capsules. Stable hydrogen isotope measurements of samples and standards were performed on hydrogen (^2^H) derived from high-temperature (1350°C) flash pyrolysis of wing tissue and continuous flow-isotope ratio mass spectrometry (CF-IRMS). Pure H_2_ was used as the sample analysis gas and the isotopic reference gas. A Hekatech HTO with an autosampler was used to automatically pyrolyse wing tissue samples to a single pulse of H_2_ gas (and N_2_ and CO gases). The resolved H_2_ sample pulse was then introduced to the isotope-ratio mass spectrometer (Elementar Isoprime (Elementar Americas, New Jersey)) via an open split capillary. Stable hydrogen isotopic ratios (^2^H/^1^H) of monarch wing tissue are reported in delta (δ) notation in parts per thousand (‰) deviation from the VSMOW-SLAP standard scale (Vienna Standard Mean Ocean Water-Standard Light Antarctic Precipitation) where δD = [(Ratio_sample_/Ratio_standard_)−1×1000]. In order to determine the δD of non-exchangeable H_2_, we used the comparative equilibration method of Wassenaar and Hobson [13] which used keratinous lab standards that had previously been calibrated for δD of non-exchangeable H_2_. Repeated within-run analyses of isotope laboratory standards yielded an analytical precision better than ±2.0‰ and ±0.3‰. The standards used for δD analysis and their within-analysis run precisions were CFS = −147.4±0.79‰ (*n* = 5), CHS = −187±0.56‰ (*n* = 5) and BWB = −108±0.33‰ (*n* = 5).

### Assignment test

Stable-hydrogen isotopes are used as a marker for estimating the origin of animals in eastern North America because weighted mean growing-season δD values in precipitation (δD_P_) vary predictably along latitudinal gradients [14], [15]. The δD values of precipitation are translated up the food web to consumer tissues [12], thereby providing a unique geospatial fingerprint, particularly in fixed (metabolically inactive) tissues. Monarch wing chitin retains the isotopic fingerprint from the time that an individual emerges from the chrysalis for the duration of its lifespan [12] and so δD values can be used to estimate natal origin using a continental δD_P_ isoscape.

To estimate natal geographic origin, we used the Online Isotopes in Precipitation Calculator [15], [16] to derive a model mean and SD of δD_P_ for each of three pre-defined breeding regions (Gulf Coast [GC], Central [CE], Great Lakes [GL]; fig. 1). The mean and SD δD_P_ values were constrained to the growing season dates of milkweed in each region. Mean milkweed emergence dates in 2009 in each breeding region were obtained from the citizen science program, Journey North [17].

In field-rearing experiments of monarchs, Hobson et al. [12] found that δD_P_ values of H_2_ used to grow milkweed are strongly correlated with the δD values incorporated into the wings of the monarchs (δD_m_) that feed on milkweed (*R* ^2^ = 0.69) according to the linear function: δD_m_ = 0.62δD_p_-79‰. We used the intercept of this function to adjust the δD_P_ values for each breeding region to be equivalent δD_m_ values [12].

Assuming a normal distribution of δD values within each region, monarchs of unknown origin were assigned a probability of origin for each of the three breeding regions based on the likelihood function:
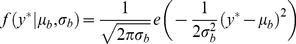
(1)Where y* = δD_m_ and μ and σ are the mean and standard deviation of each breeding region calculated from the OIPC data as described above. Likelihood values for each monarch were normalized to generate a probability of assignment for a given breeding region relative to the sum of all other breeding regions:
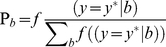
(2)Hence, each captured monarch had a probability of originating from each breeding region, with P_b_* being the region with the highest probability. We also incorporated analytical error associated with δD measurements [18] by randomly re-sampling each individual of unknown origin 100 times assuming a normal distribution with the mean equal to its δD value and the SD based on a 6 month running standard deviation of a control keratin reference (±3.3‰). We considered an individual to have originated from the breeding region which had the greatest number of P_b_* designations from the 100 re-samples (see table 3). In the results, we report the distribution of final P_b_* values for each individual, which provides a general measure of the confidence in these assignments.

**Table 3:** Confidence in assignment tests of monarchs captured throughout the Great Lakes region where each monarch was re-sampled 100 times and assignments were determined based on the highest P_b_ * value. Stable isotope value from each monarch were resampled 100 times.

assignment confidence						
capture state or province	sample size (*n*)*	≥0.9	≥0.8	≥0.7	≥0.6	≥0.5
Ontario	31	28%	58%	69%	86%	100%
Michigan	22	0%	18%	64%	82%	100%
Wisconsin	50	30%	52%	74%	88%	100%
Minnesota	30	20%	53%	70%	93%	100%
Total	133	23%	49%	71%	88%	100%

* stable isotope values from each monarch were re-sampled 100 times.

Confidence in assignments represent the proportion of 100 re-samples for each monarch that were estimated to have originated from the same region based on subsequent assignments. The percentage of monarchs that are assigned to a breeding region with a given confidence (>0.5–>0.9) is shown for each state/province.

## Results

Overall, the confidence of assignment tests was high. Of the 133 individuals that were resampled 100 times each (*n* = 13300), 12% had P_b_* values (probability of originating from the assigned breeding region) >0.9 probability, 25% had 0.8 or higher, 52% had 0.7 or higher, and only 2% had P_b_* values lower than 0.5. Summarizing the 100 re-samples for each monarch, 23% had P_b_* assignments to the same region greater than 90% of time, 71% had >70% in the same region, and none had P_b_* assignments to the same region less than 50% of the time (table 3).

Based on the assignment tests, we estimated that 36 monarchs originated from the Gulf Coast (GC) region and hence were considered to be 1^st^ generation individuals utilizing the SB strategy (see table 1 for predictions). Of the 68 monarchs originating from the Central (CE) region, 59 had wing wear scores <4 and were, therefore, considered to have followed the SB strategy (table 1). The remaining 9 individuals that were born in the CE region had wing wear scores ≥4, consistent with predictions that they followed the SS strategy (table 1). Of the 29 individuals that were estimated to have originated from the Great Lakes (GL) region, 2 had wing wear scores ≥4, consistent with the SS strategy and the remaining 27 individuals were offspring born in the GL region that were from either overwintering or 1^st^ generation individuals from further south. In total, 90% (*n* = 95) of monarchs that were not offspring born in the GL region followed the SB strategy and 10% (*n* = 11) followed the SS strategy. Surprisingly, of those that followed the SB strategy, 38% were from the GC region and 62% were from the CE region.

## Discussion

We provide evidence that monarchs adopt one of two distinct migratory strategies during the northward migration [10]. We estimated that the majority (∼90%) of monarchs re-colonizing the Great Lakes region during spring are 1^st^ generation individuals born in the Gulf Coast and Central regions of the U.S.A. (successive brood: SB) but that a small proportion of individuals (∼10%) originate directly from Mexican over wintering sites (single sweep: SS). Our estimate of the proportion of SB individuals (90%) is in remarkable agreement with that reported by Malcolm et al. [10], despite the fact that they used a different chemical marker (cardenolides) to estimate natal origin. Macolm et al. [10] also speculated that the remaining 10% of individuals that they could not identify as SB monarchs likely adopted a SS strategy and our results for SS monarchs (10%) show that this is likely the case.

Our assignment of migration strategy was based on a combination of stable isotope values and wing wear. Although the confidence in assignments to specific breeding regions based on stable isotope values was high, it is possible that some monarchs were mis-assigned to have been born in the previous year or sampling year due to some uncertainty in the relationship between wing wear and age. Of the monarchs estimated to have originated from the Gulf Coast region, 75% (n = 27/36) had wing wear scores within the expected range (1–3). However, it is unlikely that the 25% (n = 9) of Gulf Coast monarchs with wing wear ≥4 were mis-assigned since milkweed does not grow in the Gulf Coast during late summer so they could not have been born there the previous year. The most likely opportunity for a year mis-assignment existed with monarchs originating from the Central region. However, the phenology of monarch arrival in this region and wing wear data suggests that the probability of error is likely low. The earliest observation of monarch eggs within the Central region during the spring of 2009 was on April 4 at the extreme southern edge of this area [17], [22]. With an average generation time of 47 days (based on degree day calculations: NGM, DRN *unpublished data*), we estimated that monarchs born in this region would be no older than 24 days (given average sampling date for Central monarchs with wing wear ≥4 was June 14). This is likely insufficient time for monarchs to develop a high degree of wear, particularly if we consider that individuals returning from over wintering sites that were sampled on the Gulf Coast had wing wear scores of 3.28 (±0.83) and monarchs that were sampled in the Great Lakes region that were born in the Gulf Coast the same year had wing wear scores of 2.94 (±1.16) [10]. We certainly do not dismiss the possibility that some young monarchs may have had high wing wear scores due to chance events but there is no evidence for the frequency of these events so it is not possible to estimate error rates. Furthermore, the current evidence outlined above is fairly consistent with the assumption that wing wear is a reasonably good estimate of whether individuals overwintered in Mexico or were born during the sampling year.

It is possible that we underestimated the proportion of monarchs adopting a single sweep strategy because our sampling took place only once at each site during the spring. Hence, monarchs originating from over wintering sites could have died soon after arrival but prior to our sampling. We ran a simulation model to examine this possibility (NGM, DRN *unpublished data*) and found that SS monarchs may account for as much as 25% of monarchs arriving in the Great Lakes region during spring. Hence, SS monarchs could contribute a significant proportion of offspring to this area during June and early July but more thorough sampling throughout the entire spring period would help resolve this uncertainty.

Regardless of the exact proportion, it is likely that each of these re-colonization strategies have distinct costs and benefits. Single sweep individuals migrating directly north from Mexico to the Great Lakes region during early spring are able to exploit the first emerging milkweed plants, which likely gives them a significant advantage over individuals arising from the SB strategy, who must go through an additional generation before migrating north. Many of the offspring of SS monarchs have already emerged and begun laying eggs upon the arrival of SB monarchs. In addition, individuals laying eggs further north may experience lower rates of parasitism by *Ophryocystis elektroscirrha*, a protozoan parasite transmitted from adult monarchs to eggs that can cause high mortality rates [19]. Altizer et al. [20] found that over 98% of monarchs reared on *A. curassavica* in Florida were infected with *O. elektroscirrha* in comparison with less than 4% in monarchs reared on *A. syriaca* in Wisconsin and Minnesota. Hence, eggs from single sweep monarchs, which are likely deposited over a range of latitudes, may have lower overall incidence of parasitism compared to SB monarchs that lay all of their eggs at southern latitudes. However, the benefits of early arrival in the north are likely balanced with a slower maturation time of larvae and a high mortality risk associated with the unpredictable, often freezing temperatures in the spring. Hence, the proportions of monarchs from each strategy observed in the Great Lakes region during spring and early summer likely vary among years depending on the spring weather conditions along the migratory route. In years when temperatures in the Great Lakes are warmer than average, it is likely that a larger proportion of monarchs would employ a SS migration strategy compared with colder years (e.g spring 2009; [21], [22]) when the costs would outweigh the benefits and prevent early migration to this region. Thus our results should be treated with some caution because our sampling was conducted over a single season.

It is possible that these strategies are also influenced by wind patterns, as there are some anecdotal records of large numbers of monarchs arriving in the American Midwest and northern areas of the breeding range during periods of strong southern winds [7]. Although winds may act to facilitate northern migration of SS monarchs, it is unlikely that it plays a significant role in determining the strategies that individuals employ because evidence suggests that monarchs are able to decide when to migrate, often waiting until wind conditions are optimal [23].

In contrast to previous work that suggests that the majority of monarchs arriving in the Great Lakes region in spring originate from south of 35°N (10 Malcolm et al. 1993), we found that 64% of individuals (68/106) were born between 35°N–41°N in the Central part of the eastern U.S.A. (Central region). Of these, 62% of 1^st^ generation SB monarchs born during the current year (59/95) and 82% of all over wintering SS monarchs born the previous year (9/11) were born in the CE region. This also suggested that SS monarchs were migrating further than 35°N to produce a large cohort of SB monarchs in the Central region and that this area is likely an important breeding ground for fall migrants travelling to Mexico in late summer. This area of high monarch productivity corresponds with the results of Wassenaar and Hobson [24], who used stable-isotopes to show that ∼50% of monarchs overwintering in Mexico were from this region of the central U.S.A.

Previous work has suggested that conservation efforts aimed at protecting habitat in the Gulf Coast may be particularly important for monarchs following a SB migration strategy since these areas are thought to be the primary breeding grounds of this life-history strategy [10]. However, our work suggests it will also be important to protect habitat in the Central region as this area produces 62% of all SB monarchs that travel to the Great Lakes during spring. Areas north of the Gulf Coast are also particularly important for breeding monarchs since milkweed south of 35°N senesces rapidly during May, and becomes unavailable to monarchs during mid to late summer [11]. Hence, our results suggest that conservation efforts aimed at monarch habitat restoration are best targeted towards the highly productive central or mid-west region between 35°N–41°N. That said, conservation actions directed towards particular regions should be treated with caution as proportions could vary annually depending on weather conditions.

Despite the long-standing mysteries surrounding the spring migration of the monarch, even less is known about the migrations of many other insect species of dragonflies, moths, butterflies, and aphids that also migrate north during the spring. Understanding such migrations is even more important when considering that many of the major insect pests which cause billions of dollars of damage to the agricultural industry each year are also migratory [3]. Agricultural pests such as aphids and beetles travel north each spring by exploiting northward moving weather fronts which assist in dispersing individuals over a wide breeding range [25]. However, the natal origins of such economically costly insects remain unknown, and without identifying how breeding populations are spatially connected throughout the annual cycle it is very difficult to know where to allocate resources devoted to managing such pests [5]. As we have shown, the use of stable isotopes could help resolve such mysteries by estimating natal origins of individuals, and hence, the techniques presented here are useful in managing pest species as well as protecting populations at risk of extinction.

Our research helps to resolve the degree to which individuals utilize different migration strategies to re-colonize eastern North America each spring. In doing so, we provide critical information needed for conservation planning of this species by identifying important breeding locations for the monarch during the northward migration from Mexico.
